# Supplementary material for: Barriers and facilitators of adherence to long-term antiretroviral treatment in Kampala, Uganda
Source: PLOS Glob Public Health. 2025 Mar 13;5(3):e0004121. doi: 10.1371/journal.pgph.0004121 (PMC11906038; doi:10.1371/journal.pgph.0004121)
Supplement: Attachment — Submitted filename: Response to editorial comments.docx [file pgph.0004121.s002.docx]

**7^th^ October 2024**

Jan Louis Adajo Non

PLOS Global Public Health

Dear Jan,

**RE: RESPONSE TO EDITORIAL COMMENTS**

1. Please report in the Methods section the CORRECT start and end date of the recruitment period for this study in DD/MM/YYYY format. Please note that if this information is not included when your manuscript is resubmitted, it may be rejected

**Response**: Thank you for the comment. I have inserted the correct start and end dates in the methods section of the abstract as **January and June 2022** **(Line 32).** I have also inserted the DD/MM/YYYY in the methods section of the manuscript as “**from 3^rd^ January to 30^th^ June 2022”**. **Line 101**

2. Please upload a copy of the original ethics approval letter from National Council Uganda as required by question 1 of the human subject research checklist. If the original letter is not in English, please also provide an English-translated version in the supporting file. Please note that if this documentation is not included when your manuscript is resubmitted, it may be rejected.

**Response**: Thank you for the comment. I have uploaded the Uganda National Council for Science and Technology approval letter as guided

3. You indicated that you had ethical approval for your study. In your Methods section, please ensure you have also stated whether you obtained consent from parents or guardians of the minors included in the study or whether the research ethics committee or IRB specifically waived the need for their consent.

**Response:** Thank you for the comment. This study obtained consent from adults who could make informed consent. No Minor was enrolled in this study. Under ethical approval (Line **142).** We have stated that we obtained consent from the participants since our inclusion criteria were for participants above 18 years (**Line 110)**

Thank you,

Okoboi Stephen
