# Supplementary material for: Barriers and facilitators of adherence to long-term antiretroviral treatment in Kampala, Uganda
Source: PLOS Glob Public Health. 2025 Mar 13;5(3):e0004121. doi: 10.1371/journal.pgph.0004121 (PMC11906038; doi:10.1371/journal.pgph.0004121)
Supplement: Attachment — Submitted filename: Responses to Reviewers_11_12_24.docx [file pgph.0004121.s003.docx]

**11^th^ December 2024**

Janet Seeley

Academic Editor

PLOS Global Public Health

Dear Janet,

**RE: RESPONSE TO REVIEWERS COMMENTS**

I sincerely thank the reviewers for their insightful comments, which will improve the quality and clarity of the manuscript.

Please find below our response to the reviewers and editorial comments on manuscript number PGPH-D-24-02254.

**Editorial comment 1:** Journal Requirements:

Please include a complete copy of PLOS’ questionnaire on inclusivity in global research in your revised manuscript. Our policy for research in this area aims to improve transparency in the reporting of research performed outside of researchers’ own country or community. The policy applies to researchers who have travelled to a different country to conduct research, research with Indigenous populations or their lands, and research on cultural artefacts. The questionnaire can also be requested at the journal’s discretion for any other submissions, even if these conditions are not met. Please find more information on the policy and a link to download a blank copy of the questionnaire here <https://journals.plos.org/globalpublichealth/s/best-practices-in-research-reporting>. Please upload a completed version of your questionnaire as Supporting Information when you resubmit your manuscript.

**Response:** Thank you for the PLOS questionnaire on inclusivity in global research. I downloaded and reviewed the form carefully. Based on the review, I confirm that we are ineligible to complete it now. However, we are happy to be guided further.

**Editorial Comment 2:** Please review your reference list to ensure that it is complete and correct. If you have cited papers that have been retracted, please include the rationale for doing so in the manuscript text, or remove these references and replace them with relevant current references. Any changes to the reference list should be mentioned in the rebuttal letter that accompanies your revised manuscript. If you need to cite a retracted article, indicate the article’s retracted status in the References list and also include a citation and full reference for the retraction notice.

**Response:** Thank you for this editorial comment. We have reviewed our reference list and confirm it is complete and correct. We added reference 24 **(Hennink M, Kaiser BN. Sample sizes for saturation in qualitative research: A systematic review of empirical tests. Soc Sci Med. 2022 Jan; 292:114523. doi: 10.1016/j.socscimed.2021.114523. Epub 2021 Nov 2. PMID: 34785096)**, which cites evidence that 9-17 interviews can reach thematic saturation to justify our study's selection of 22 key informants. As requested, references have been renumbered.

**Reviewer 2, comment 1**: Explain why you decided to interview 16 PLWH and 6 HCPs. You mention in the paper that you reached data saturation but it is not clear why you decided on these numbers initially and whether you stopped interviewing based on saturation - that said saturation is increasingly being challenged in qualitative research as an arbitrary justification for qualitative sample size decisions. Therefore, I would prefer to know why you decided to interview this number of participants in the first place.

**Response:** Thank you so much for this critical comment. Twenty-two participants (16, PLWH, and six healthcare providers) were chosen based on the literature indicating that 9 -17 key interviews and 4-8 focus group discussions could reach saturation of themes **(Hennink M, Kaiser BN. Sample sizes for saturation in qualitative research: A systematic review of empirical tests. Soc Sci Med. 2022 Jan; 292:114523. doi: 10.1016/j.socscimed.2021.114523. Epub 2021 Nov 2. PMID: 34785096).**

We interviewed all participants as planned and approved by the regulatory bodies. For healthcare workers, the study also purposively included those who had provided ART services for at least three years but were primarily dedicated to managing ALT cohort participants as described under **sample size and data collection, lines 112-115**. We have clarified this further to read**, “Based on the literature that 9 -17 interviews can reach saturation [24], we purposively sampled 22 participants. Of the 22 participants, 16 were PLWH, and six were healthcare providers” (lines 109-110).**

In addition, saturation has roots in grounded theory and has gained momentum and recognition over the years to enhance robustness in qualitative research. Despite increasing acceptance, it has been controversial because of difficulties with definition, nature, purpose, and usage variations. There are three main definitions of saturation: code saturation, thematic saturation, and meaning saturation, which are related to variations in qualitative research designs, data collection methods, and instruments. In our study, we used thematic saturation “lines **122-123 of the manuscript”** based on **(Sebele-Mpofu, F. Y. (2020). Saturation controversy in qualitative research: Complexities and underlying assumptions. A literature reviews. Cogent Social Sciences, 6(1). https://doi.org/10.1080/23311886.2020.1838706**

**Reviewer comment 2:** You use the term "PLWH interviews" - it would be preferable to talk about 'interviews with PLWH."

**Response:** Thank you so much for picking this up. We agree with the suggestion to use people-first language and have revised the manuscript to use the term “interviews with PLWH.”

Thank you,

Okoboi Stephen

Corresponding author
